# Platelet Function Tests: A Review of Progresses in Clinical Application

**DOI:** 10.1155/2014/456569

**Published:** 2014-05-08

**Authors:** Jae-Lim Choi, Shuhua Li, Jin-Yeong Han

**Affiliations:** Department of Laboratory Medicine, Dong-A University College of Medicine, 1,3-Ga, Dongdaesin-dong, Seo-gu, Busan 602-715, Republic of Korea

## Abstract

The major goal of traditional platelet function tests has been to screen and diagnose patients who present with bleeding problems. However, as the central role of platelets implicated in the etiology of arterial thrombotic diseases such as myocardial infarction and stroke became widely known, platelet function tests are now being promoted to monitor the efficacy of antiplatelet drugs and also to potentially identify patients at increased risk of thrombosis. Beyond hemostasis and thrombosis, an increasing number of studies indicate that platelets play an integral role in intercellular communication, are mediators of inflammation, and have immunomodulatory activity. As new potential biomarkers and technologies arrive at the horizon, platelet functions testing appears to take on a new aspect. This review article discusses currently available clinical application of platelet function tests, placing emphasis on essential characteristics.

## 1. Introduction

Platelets are small, anucleated cytoplasmic bodies circulating in blood stream. These cellular fragments are derived from megakaryocytes in the bone marrow. In steady state, megakaryocytopoiesis supplies about 10^11^ platelets per day with a new turnover every 8-9 days. This process is influenced by various environmental changes and platelets normally circulate at concentrations of 150–400 × 10^9^/L [1, 2].

Resting platelets appear small discoid cells (2–4 *μ*m by 0.5 *μ*m), facilitating their margination toward the vessel wall, where they can constantly survey the integrity of the vascular endothelium. Platelets contain three major types of granules: *α*-granules, dense bodies, and lysosomes. *α*-Granules are the most abundant granules in platelets and are rapidly exocytosed upon activation to enhance hemostasis and inflammation. Dense bodies contain adenine nucleotides (ADP and ATP) and serotonin which induce platelet aggregation, vasoconstriction, cytokine production, and modulators of inflammation. Lysosomes contain glycohydrolases and proteases that can aid in pathogen clearance, breakdown of extracellular matrix, and contribute to the clearance of platelet thrombi and degradation of heparin [1–3].

The normal vascular endothelium produces potent platelet inhibitors such as nitric oxide, prostacyclin, and natural ADPase. Once subendothelial components including collagen, fibronectin, laminin, or von Willebrand factor (vWF) become exposed upon vessel wall injury, platelets undergo a highly regulated series of functional reactions like adhesion, spreading, release reaction, aggregation, procoagulant activity, microparticle formation, and subsequently clot retraction. Adhesion is mediated by the interaction between the glycoprotein (GP) Ib/V/IX receptor complex on the platelet surface to vWF and GP VI and GP Ia to collagen at the sites of vascular injury [3–5].

Multiple pathways bring to platelet activation such as collagen, ADP, thromboxane A2, epinephrine, serotonin, and thrombin. The cumulative action of these activators results in recruitment of platelets from the circulation and several distinct manifestations of platelet activation including platelet shape change, expression of P-selectin, soluble CD40 ligand and platelet procoagulant activity, and conversion of GP IIb/IIIa into an active form. GP IIb/IIIa is the central platelet receptor mediating platelet aggregation. Only the activated GP IIb/IIIa complex is able to bind soluble plasma fibrinogen leading to ultimate aggregation and further spreading of the stimulated platelets along the site of injury. Therefore, aggregation is critically dependent on G-protein coupled receptors and is mediated by bridges between fibrinogen/vWF (under high shear) and the activated GP IIb/IIIa complexes on adjacent, stimulated cells [1–6]. The exposure of anionic phospholipids, mainly phosphatidylserine, provides a surface upon which platelets can support thrombin. Thrombin, the key enzyme of the coagulation cascade and the most potent platelet agonist, acts by cleaving protease-activated surface receptor. The resulting thrombin burst leads to further activation and local recruitment of platelets into the vicinity and inclusion of leukocytes via their receptors for P-selectin. After the clot has been formed, the activated platelets rearrange and contract their intracellular actin/myosin cytoskeleton, which results in clot retraction.

Most platelet function tests have been traditionally used for the diagnosis and management of patients presenting with bleeding problems. In contrast to coagulation defects, where screening tests such as prothrombin time (PT) or activated partial thromboplastin time (aPTT) are more standardized and fully automated, platelet function tests are still labor intensive and time-consuming and require special equipment and experts of specialized laboratories. The laboratory identification of hemostasis defects including platelet function disorders now involves a multistep process. Because platelets are implicated in atherothrombosis as well, newer and existing platelet function tests are increasingly used for monitoring the efficacy of antiplatelet drugs, with the aim of predicting the adverse events such as bleeding or thrombosis in arterial thrombotic diseases. This review article discusses currently available clinical application of platelet function tests, placing emphasis on essential characteristics.

## 2. Platelet Function Testing

When investigating platelet function disorders, a stepwise process is required, and collaboration between clinical and laboratory personnel is important to obtain a detailed history. A review of recent and regular medications is also required. Then laboratory testing is initiated, which begins with a full blood count, often in conjunction with a blood film examination, particularly if the hematology analyzer gives abnormal platelet flags regarding the platelet count, mean platelet volume (MPV), or platelet distribution width (PDW). Many laboratories are utilizing the panel of screening tests when a patient presents with a clinical suspicion of defects in hemostasis. If the screening tests are all normal but the clinical indication is strong for platelet defects, it is imperative that a complete diagnostic workup is still performed. If appropriate, a complex panel of specialized tests including platelet aggregometry, a measure of platelet release, flow cytometry, platelet microRNAs (miRNA), genetic studies, and analysis of signal transduction pathways will be done.  Table 1 provides a summary of currently available platelet function tests with their clinical utility [2, 7–10].

### 2.1. Platelet Aggregometry

Light transmission aggregometry (LTA) is regarded as the gold standard of platelet function testing and is still the most used test for the identification and diagnosis of platelet function defects. Platelet rich plasma (PRP) is stirred within a cuvette located between a light source and a detector. After addition of a various panel of agonists, such as collagen, ADP, thrombin, ristocetin, epinephrine, and arachidonic acid, the platelets aggregate and light transmission increases. Thrombin is a potent platelet agonist. But thrombin cleaves fibrinogen and leads clot formation. It is a difficult agonist to use for platelet aggregation testing. Instead of thrombin, thrombin receptor activating peptide (TRAP) is used for thrombin receptors. The platelet aggregation pattern is thought as a primary response to an exogenous agonist, followed by a secondary response to the release of dense granule contents. This biphasic response can be masked if high concentrations of agonists are added. Parameters measured include the rate or slope of aggregation (%/min) and the maximal amplitude (%) or percentage of aggregation after a fixed period of time, usually 6–10 min [7–12].

Traditional LTA remains the most useful technique for diagnosing a wide variety of platelet defects. The main disadvantage of LTA is the use of PRP instead of the whole blood under relatively low shear conditions, and, in the absence of red and white cells, it does not accurately simulate primary hemostasis. It also requires large sample volume and is time-consuming and there are many preanalytical and analytical variables that affect the LTA results. The LTA technique is not standardized, despite the fact that guidelines have been published. Recent recommendations from the Platelet Physiology Subcommittee of the Scientific and Standardization Committee (SSC) of the International Society on Thrombosis and Haemostasis (ISTH) are available [13–15]. Alternative methods including whole blood aggregometry or lumiaggregometry have been introduced, but the majority of these techniques have not been widely adopted and failed to provide additional diagnostic information.

The limitations of conventional LTA assay triggered the development of new, easier-to-use platelet function tests. The most widely used test today is the platelet function analyzer- (PFA-) 100 and PFA-200 devices (Siemens, Marburg, Germany), which is considered to be a surrogate in vitro bleeding time. The instrument monitors the drop in flow rate, and the time required to obtain full occlusion of the aperture is reported as closure time (CT), up to a maximum of 300 seconds. This global platelet function test is easy to use, automated, and rapid and mimics several characteristics of physiologic platelet function, because it is a high shear system and the whole blood is used instead of PRP. Clinical applications have been recently reviewed, and they include screening for von Willebrand disease (vWD) and its treatment monitoring, identification of inherited and acquired platelet defects, monitoring antiplatelet therapy, and assessment of surgical bleeding risk [16, 17]. However, the CT is influenced by platelet count and hematocrit, and they would have poor specificity for any particular disorder. Thus, normal PFA CT results can be used with some confidence to exclude severe vWD or severe platelet dysfunction but would not exclude a possible mild vWF deficiency or mild platelet disorder. Any presumptive platelet function defect detected by abnormal CT needs to be confirmed by more specific tests.

### 2.2. Flow Cytometry

Whole blood flow cytometry is a powerful and popular laboratory technique for the assessment of platelet function and activation. Although flow cytometry requires sophisticated equipment, requires available monoclonal antibodies, and is not well-standardized, it has several advantages including small volume of whole blood and independence of platelet count. The most commonly used routine flow cytometry tests are the quantification of the basal platelet GP receptor status and the determination of the platelet granule composition. Flow cytometer can quantify GP IIb/IIIa deficiencies in Glanzmann's thrombasthenia and GP Ib/IX/V in Bernard-Soulier syndrome. It has also been devised to measure dense granules using mepacrine uptake and release [2, 7–12]. Flow cytometry allows the analysis of individual platelet functional capability and the measurement of the expression of platelet activation markers on individual platelets as well as the quantitation of associates between platelets and other blood cells.

The most common platelet activation markers assessed by flow cytometry are P-selectin expression on the platelet surface (as a marker of *α*-granule secretion), the conformational change of GP IIb/IIIa into its active state (measured with monoclonal antibody PAC-1), platelet-leukocyte conjugates, microparticle examination, exposure of anionic, negatively charged phospholipids on the platelet surface (procoagulant activity), and phosphorylation of vasodilator-stimulated phosphoprotein-phosphorylation (VASP-P) (BioCytex, Marseille, France), as a marker of P2Y12 receptor activation-dependent signaling [18–20]. Many laboratories have measured a variety of different platelet activation markers and shown that they are elevated in various clinical conditions such as unstable angina, acute myocardial infarction, preeclampsia, peripheral vascular disease, and cerebrovascular ischemia.

### 2.3. Other Point-of-Care Testing (POCT)

Platelet function tests are increasingly proposed as perioperative tools to aid in prediction of bleeding or for monitoring the efficacy of various types of prohemostatic therapies. As increasing numbers of patients are being treated with antiplatelet drugs, there is also an associated increased risk of bleeding. Traditional LTA still has an important role in the evaluation of platelet disorders, but it cannot easily be performed in the acute care conditions. The growing clinical need coupled with the development of new, simpler POCT machines has resulted in a tendency of platelet function tests to be done away from specialized clinical hemostasis laboratories. This review focuses on the clinical utility of two representative instruments: impact cone and plate analyzer (DiaMed, Cressier, Switzerland) [2, 18, 19] and thrombelastography (TEG) (Hemoscope, Niles, IL, USA) [7–10, 21, 22] in more detail, and the others are referred to below in the section of monitoring antiplatelet therapy.

The impact cone was originally designed to monitor platelet adhesion to a polystyrene plate. The instrument contains a microscope and performs staining and image analysis of the platelets that adhere and aggregate under a high shear rate of 1,800/sec. The results are reported as the percentage of the surface covered by platelets (surface coverage) and the average size of the adherent particles. The adhesion is dependent on vWF, fibrinogen binding, and platelets GP Ib and IIb/IIIa. The assay is fully automated, simple, and rapid to use, requiring small whole blood. Emerging data suggest that the impact cone can detect numerous platelet defects and vWD and could also be potentially used as a screening method. But the fully automated version of impact cone has limited use and therefore further studies are required [2, 7–10, 18, 19].

TEG and rotational thrombelastometry (ROTEM) (Pentapharm GmbH, Munich, Germany) measure the physical properties of forming clots by the use of an oscillating cup that holds whole blood samples. TEG provides various data related to fibrin formation, clot development, the ultimate strength, and stability of fibrin clot as well as fibrinolysis. The particular advantages of TEG/ROTEM are to provide a complete profile of clot formation and allow for interactions between whole blood elements, including platelets and the coagulation system. Unlike other specific platelet function tests, these instruments have traditionally been used within surgical and anesthesiology departments for determining the risk of bleeding and as a guide for transfusion requirements. There is interlaboratory variation and it is a time-consuming analysis (at least 30 min) [7–10, 21, 22].

## 3. Monitoring Antiplatelet Therapy

As combined antiplatelet regimens become more widely used and newer agents offer more potent antiplatelet effects, it will become increasingly important to be able to optimize the risk-to-benefit ratio in individual patients. Modern antiplatelet therapy based on the inhibition of three major platelet activation pathways, (1) cyclooxygenase-1 inhibition resulting in a reduction of thromboxane A_2_, (2) P2Y12 (ADP) inhibition, and (3) GP IIb/IIIa receptor blockade, is a cornerstone in the successful treatment [23–25]. Although dual therapy with aspirin and clopidogrel has proven to positively influence outcome in patients with acute coronary syndrome and, after percutaneous coronary interventions (PCI), a considerable variation in individual response to antiplatelet therapy assessed by different techniques can be observed [23–27]. These findings may be explained by the different testing populations but may primarily be a result of interassay variations such as different sensitivities, differences in platelet activation techniques, and variations in type of activators and their concentrations.

In the past decade, compelling evidence from numerous observational studies has emerged demonstrating a strong association between high platelet reactivity (HPR) to ADP and post-PCI ischemic events, especially stent thrombosis. In 2010, the Food and Drug Administration (FDA) added a boxed warning to clopidogrel to alert prescribers to the possibilities of CYP genetic polymorphisms that can result in poor metabolism and poor clopidogrel responsiveness [28]. The American Heart Association/the American College of Cardiology Foundation and the European Society of Cardiology guidelines issued a class IIb recommendation for platelet function testing to facilitate the choice of P2Y12 inhibitor in selected, high-risk patients undergoing PCI, although routine testing is not recommended (class III). Recently they updated the consensus document and proposed updated cutoff values for HPR and low platelet reactivity (LPR) to ADP that might be used in future investigations of personalized antiplatelet therapy. LPR to ADP is suggested to be associated with a higher risk of bleeding. Therefore, they proposed a therapeutic window concept for P2Y12 inhibitor therapy [29–31].

The most widely used platelet function assays, VerifyNow P2Y12 assay (Accumetrics, San Diego, CA, USA) and, at present, Multiplate analyzer (H. Hoffmann-La Roche Ltd., Basel, Switzerland) and VASP assay, have overcome many of the technical and methodological limitations of the previous assays, including conventional LTA. Thus, at the present time, HPR and LPR in the setting of PCI have been defined by the receiver-operator characteristic (ROC) curve analyses using the following criteria, respectively: (1) >208 and <85 P2Y12 reaction units (PRU) by VerifyNow P2Y12 assay, (2) >50% and <16% platelet reactivity index (PRI) by VASP-P, and (3) >46 and <19 arbitrary aggregation units (AU) in response to ADP by Multiplate analyzer [29–31]. However, there are no large-scale clinical studies to date demonstrating that the adjustment of antiplatelet therapy based on any of these cut points improves clinical outcome. Currently, platelet function testing may be considered in determining an antiplatelet strategy in patients with a history of stent thrombosis and in patients prior to undergoing high-risk PCI [32–34].

## 4. Platelet-Derived Microparticles (PMP)

Circulating microparticles (MP) are defined as small and anucleoid phospholipid vesicles, approximately 0.1–1.0 *μ*m in diameter, and derived from different cell types such as platelets, erythrocytes, leukocytes, endothelial cells, and vascular smooth muscle cells [35]. MP are distinguished from exosomes, which are smaller vesicles (40–100 nm) derived from endoplasmic membranes and apoptotic bodies that are larger particles (>1.5 *μ*m) and contain nuclear components. MP carry surface proteins and include cytoplasmic materials of the parental cells, while MP membrane includes negatively charged phospholipids, mainly phosphatidylserine, thus, responsible for the exertion of microparticle-mediated biological effects [36, 37].

Under steady-state conditions, MP originating from platelet and megakaryocytes are the most abundant microparticles, constituting up to 70–90% of all MP in circulation [38]. PMP levels show gender-specific differences, with increased numbers in women as compared with men, which is further modulated by the menstruation cycle. Increases in PMP levels are observed with aging, during pregnancy, and after exercise, leading to a concomitant increase of hemostatic potential. About 25% of the procoagulant activity of stimulated platelet suspensions is associated with MP released upon platelet activation and their surface may be approximately 50–100 times more procoagulant than the surface of activated platelet per se. Low amounts of PMP are continuously shed from platelets, but this process is highly accelerated after platelet activation or intensive physical activity.

The thrombogenic properties of PMP have been confirmed in experimental studies and high PMP levels are strongly associated with different thrombotic conditions. PMP were also significantly increased in patients with acute pulmonary embolism and were the main source of procoagulant MP. For example, in valvular atrial fibrillation, which carries a very high risk of thromboembolism, the numbers of PMP were more than threefold. Elevated PMP levels are associated with other disease states including heparin-induced thrombocytopenia, arterial thrombosis, idiopathic thrombocytopenic purpura, thrombotic thrombocytopenia, sickle cell disease, uremia, malignancy, and rheumatoid arthritis. PMP have also been implicated in the pathogenesis of atherosclerosis as well as the regulation of angiogenesis [39–43].

A number of studies indicate that MP contribute to intercellular communication. Recent studies provided now a rationale for the concept of MP as vehicles for intercellular exchange of biological signals and information. Although the physiological significance of PMP may have been overlooked for many years, ongoing research works suggest that these tiny blebs may play an important role in the transport and delivery of bioactive molecules and signals throughout the body. MP may affect target cells either by stimulating directly via surface-expressed ligands or by transferring surface receptors from one cell to the other. Because MP engulf cytoplasm during their formation, they acquire proteins and RNA that originate from the cytosol of the parent cell. An increasing body of evidence suggests that, after attachment or fusion with target cells, MP deliver cytoplasmic proteins and RNA to recipient cells. This process can be mediated either through receptor-ligand interactions or through internalization by recipient cells via endocytosis. Although antigens found on the surface of MP and the cargo of MP resemble those of their parental cells, MP represent more than just a miniature version of the specific cell of origin [35–38].

Several laboratory methods for analysis of PMP have been published, and the most widely used method today is flow cytometry. Although there are a large number of publications on PMP, the lack of standardization makes the comparison of results between studies difficult. A previous ISTH Vascular Biology Subcommittee survey showed that about 75% of laboratories use flow cytometry to quantitate MP in clinical samples. However, a wide variety of preanalytic and analytic variables have been reported in the literature, resulting in a broad range of PMP values in platelet-free plasma of healthy subjects [44–46]. Three ISTH SSC (Vascular Biology, Disseminated Intravascular Coagulation, and Haemostasis and Malignancy) have initiated a project aiming at standardization of the enumeration of cellular MP by flow cytometer. This strategy is based on the use of fluorescent calibrated submicrometer beads, Megamix beads (BioCytex, Marseille, France), which allow the MP analysis to be reproducible.

## 5. Platelet MicroRNAs (miRNA)

miRNA is a small (21–23 nucleotides) noncoding RNA regulating approximately 60% of the mammalian protein coding genes at least in part by translational repression [47]. Although platelets lack a nucleus or genomic DNA, they are able to translate inherited mRNA into protein. In addition to inherited functional translation machinery, for example, rough endoplasmic reticulum, ribosomes, and small amount of poly(A) RNA from parent megakaryocytes, the presence of a strong correlation between platelet transcriptome and proteomic profile supports de novo translational capabilities of platelets and suggests the possibility of posttranscriptional regulation of gene expression within platelets. Interestingly, platelets inherit essential miRNA processing proteins in addition to miRNA transcripts originated from their parent megakaryocytes [47–49].

Activated platelets, as discussed above, release MP rich in growth factors or variety of effecter proteins that may exert extracellular effects. A recent report of miRNA recovery from plasma MP indicates that PMP may probably deliver platelet miRNA at the site action in cardiovascular system. Moreover, platelets-secreting miRNA may contribute to the plasma miRNA pool, which has become a great attraction for scientists searching novel biomarkers associated with various pathologic conditions [49–51]. Microarray screening revealed that miR-126, miR-197, miR-223, miR-24, and miR-21 are among the most highly expressed miRNAs in platelets and PMP. Their circulating levels actually correlated with PMP as quantified by flow cytometry. Levels of miR-340 and miR-624 were found to be significantly elevated in platelets from patients who suffer from premature coronary artery disease. Similarly, miR-28 overexpression was observed in platelets obtained from myeloproliferative neoplasms [51].

The presence of more than 750 of the roughly 2,000 known human miRNAs in platelets is quite interesting. miRNA has an established role in hematopoiesis and megakaryocytopoiesis; therefore, platelet miRNAs appear to be useful biomarkers and tools for understanding mechanisms of megakaryocyte and platelet gene expression. Because mRNA translation is the only mechanism for new protein synthesis in circulating platelets, it is expected that platelet miRNA plays a role in health and disorders of hemostasis and thrombosis.

## 6. Conclusions

Most platelet function tests have traditionally been used for the diagnosis and management of patients presenting with bleeding problems. In contrast to coagulation defects, where screening tests such as PT or aPTT are more standardized and fully automated, platelet function tests are still labor intensive and time-consuming and require special equipment and experts of specialized laboratories. The laboratory identification of hemostasis defects including platelet function disorders now involves a multistep process. Because platelets are implicated in atherothrombosis as well, newer and existing platelet function tests are increasingly used for monitoring the efficacy of antiplatelet drugs, with the aim of predicting the adverse events such as bleeding or thrombosis in arterial thrombotic diseases.

Many laboratories are utilizing the panel of screening tests when a patient presents with a clinical suspicion of defects in hemostasis. If the screening tests are all normal but the clinical indication is strong for platelet defects, it is imperative that a complete diagnostic workup is still performed. If appropriate, a complex panel of specialized tests including platelet aggregometry, a measure of platelet release, flow cytometry, PMP, platelet miRNA, genetic studies, and analysis of signal transduction pathways will be done. Beyond hemostasis and thrombosis, an increasing number of studies indicate that platelets play an integral role in intercellular communication, are mediators of inflammation, and have immunomodulatory activity. As new potential biomarkers and technologies arrive at the horizon, platelet functions testing appears to take on a new aspect.

## Figures and Tables

**Table 1:** Major platelet function tests and their clinical applications.

Name of test	Principle	Clinical applications
Platelet aggregometry	Platelet aggregation to a panel of agonists	Diagnosis of inherited and acquired platelet defects
PFA-100/200	High shear platelet adhesion and aggregation	Detection of inherited and acquired platelet defects, monitoring antiplatelet drugs
Flow cytometry	Measurement of platelet GP, secretion, MP, and activation markers by fluorescence	Diagnosis of platelet GP defects, platelet release, PMP, platelet activation markers, monitoring antiplatelet drugs
Impact	Measurement of platelet adhesion and aggregation under high shear	Detection of inherited and acquired platelet defects, monitoring antiplatelet drugs
Thrombelastography (TEG/ROTEM)	Monitoring rate and quality of clot formation	Prediction of surgical bleeding, aid to blood product usage, monitoring antiplatelet drugs
VerifyNow	Platelet aggregation	Monitoring antiplatelet drugs
Multiplate	Platelet aggregation	Monitoring antiplatelet drugs
VASP-P	Flow cytometry with phosphoprotein-phosphorylation	Monitoring P2Y12 receptor activity
Microparticles	Flow cytometry with calibrated beads	Platelet activation markers, intercellular communication

PFA: platelet function analyzer; GP: glycoprotein; MP: microparticles; PMP: platelet-derived microparticles; TEG: thrombelastography; ROTEM: rotational thrombelastometry; VASP-P: phosphorylation of vasodilator-stimulated phosphoprotein-phosphorylation.
